# A Capsule Decision Neural Network Based on Transfer Learning for EEG Signal Classification

**DOI:** 10.3390/biomimetics10040225

**Published:** 2025-04-04

**Authors:** Wei Zhang, Xianlun Tang, Xiaoyuan Dang, Mengzhou Wang

**Affiliations:** 1 School of Computer Science and Technology, Chongqing University of Posts and Telecommunications, Chongqing 400065, China; 2 School of General Education, Chongqing College of Traditional Chinese Medicine, Chongqing 402760, China; wangmz@cqctcm.edu.cn; 3 School of Automation, Chongqing University of Posts and Telecommunications, Chongqing 400065, China; 4 School of Intelligent Engineering, Chongqing College of Mobile Communication, Chongqing 401520, China; dangxiaoyuancq@foxmail.com

**Keywords:** brain computer interface, Riemann manifold, capsule decision neural network, convolution neural network, capsule neural network

## Abstract

Transfer learning is the act of using the data or knowledge in a problem to help solve different but related problems. In a brain computer interface (BCI), it is important to deal with individual differences between topics and/or tasks. A kind of capsule decision neural network (CDNN) based on transfer learning is proposed. In order to solve the problem of feature distortion caused by EEG feature extraction algorithm, a deep capsule decision network was constructed. The architecture includes multiple primary capsules to form a hidden layer, and the connection between the advanced capsule and the primary capsule is determined by the neural decision routing algorithm. Unlike the dynamic routing algorithm that iteratively calculates the similarity between primary capsules and advanced capsules, the neural decision network computes the relationship between each capsule in the deep and shallow hidden layers in a probabilistic manner. At the same time, the distribution of the EEG covariance matrix is aligned in Riemann space, and the regional adaptive method is further introduced to improve the independent decoding ability of the capsule decision neural network for the subject’s EEG signals. Experiments on two motor imagery EEG datasets show that CDNN outperforms several of the most advanced transfer learning methods.

## 1. Introduction

Brain computer interface (BCI) refers to the information channel established between the human brain and external devices to realize the information exchange between the human brain and devices [1,2]. Electroencephalogram (EEG) is the rhythmic electrical activity of neurons obtained by processing the bioelectric signals of the human brain through precise electronic instruments. Electroencephalogram (EEG) is usually a multi-channel time series, which is the most convenient research object of the brain–computer interface. The EEG-based brain–computer interface includes three common modes: motor imagery (MI) [3], event-related potentials (ERPs) [4], and steady-state visual evoked potentials [2]. The research of this paper mainly involves the brain–computer interface of motor imagery.

In the motor imagery mode, when a subject performs a motor imagery task in a certain direction, the EEG rhythmic energy in the contralateral motor sensory area of the cerebral cortex will significantly decrease, while the EEG rhythmic energy in the ipsilateral motor sensory area will increase. This phenomenon is called event-related desynchronization (ERD)/event-related synchronization (ERs). Thus, the human brain can actively control the left and right brains μ,β. The rhythmic amplitude can generate a variety of control commands. Event-related potential (ERP) is a kind of special brain-evoked potential, which is caused by a single or multiple stimuli by intentionally endowing stimuli with specific psychological meaning. The brain–computer interface system based on EEG has been widely used in auxiliary medical equipment [1].

In order to correctly identify EEG signals, the signal processing process generally includes the following three steps: preprocessing, feature extraction, and feature classification [5]. Spatial filters, such as common spatial pattern (CSP), are used to extract the most discriminating feature information [6]. In recent years, in EEG signal analysis, the representation of the EEG signal covariance matrix has been widely studied. The covariance matrix is a symmetric positive definite matrix (SPD) that can be processed by the manifold learning theory and method [7,8,9].

In the task of motor imagery EEG signal processing, the feature information is mainly spatial information, which can be directly encoded by the covariance matrix. However, the main characteristic information of ERP tasks is time–domain information to some extent, and the effect of direct coding with the covariance matrix is not ideal. In reference [10], a new method was proposed to improve classification performance by using spatial covariance that is not sensitive to ERP waveform delay and amplitude distortion.

One of the main difficulties of BCI is that the EEG signals of different subjects with the same stimulus are very different, and even the EEG signals of the same subject with the same stimulus are different in multiple trials. In order to solve this problem, the method of transfer learning is used to reduce the individual differences of EEG signals. By minimizing the differences between the source domain and the target domain, the source domain and the target domain meet the independent and identical distribution as much as possible to achieve better classification results in the target domain [11,12]. In addition, Arunabha M. Roy et al. proposed a multi-scale fusion method based on transfer learning that can effectively extract distinguishable features of non-overlapping standard frequency bands of EEG signals [13,14]. Rahul Sharma et al. proposed a novel multi-layer perceptron model that is independent of the subjects and can more robustly decode EEG signals [15]. In recent years, some representative transfer learning models have been proposed in the field of brain–computer interface [16,17,18]. C.P.A. Moraes et al. proposed an innovative method of transfer learning task that improved the correlation between subjects by exploring the application of minimum mutual information to motor imagery through independent vector analysis [19]. D. Li et al. proposed a continuous motor imagery EEG classification method based on the domain incremental learning framework, which was applied to the scene requiring continuous knowledge transfer and greatly improved the problem, which was that the performance of the transfer learning model gradually declined with the increase of the number of transfers [20]. Y. Jin et al. are committed to finding an adaptive covariance matrix to improve the robustness of classification [21].

At the same time, manifold-based transfer learning has also been widely studied. Zanini et al. [22] used the theory of Riemannian manifold to transform the covariance of different subjects to make their distribution tend towards consistency. Yair O. and his team innovatively introduced a perspective that utilizes covariance matrices as lenses to gain insights into data characteristics, and they designed a novel strategy that employs parallel transfer techniques to achieve domain adaptation on a conical manifold structure composed of symmetric positive definite matrices [23]. On the other hand, Sangineto E. et al. followed the traditional framework of transfer learning, constructing their method around a set of source topics aimed at training specific classification models for multiple subjects and then seamlessly transferring the parameter knowledge from these models to the target individual, thereby achieving effective knowledge transfer [24]. M. Islam et al. integrated spatiotemporal features to construct a CNN-LSTM hybrid model, which demonstrated excellent performance in sentiment detection tasks [25]. Veeranki Y R et al. proposed a new method to extract nonlinear features from EDA signals by using a self-encoder based on deep learning to deal with the inherent nonlinear and non-stationary characteristics of signals [26]. At the same time, in the field of emotion recognition of EEG signals, a frequency conversion complex demodulation method for obtaining a high-resolution time spectrum from EEG signals was proposed to solve the similar problems of EEG signals [27]. He and Wu [28] proposed a new method to extend Riemann alignment to European alignment in European Space in order to adapt to European classifiers.

Manifold learning is a valuable research direction in processing nonlinear EEG signals, and using the neural network method to realize the decision tree in machine learning can avoid hard boundaries. In order to make full use of the nonlinear classification ability of Riemannian manifolds and mine the transferability from the source domain to the target domain, a capsule decision neural network based on transfer learning (CDNN) is proposed in this paper. Aiming at the problem that the EEG feature extraction algorithm easily causes feature distortion, a deep capsule decision network is constructed. The architecture includes multiple primary capsules to form a hidden layer, and the connection between advanced capsules and primary capsules is determined by the neural decision routing algorithm. Unlike the dynamic routing algorithm, which iteratively calculates the similarity between the primary capsule and the advanced capsule, the neural decision network calculates the relationship between each capsule in the deep and shallow hidden layers in the way of probability decision. The neural decision component realizes the dynamic correlation between advanced capsules and primary capsules so that the model can automatically screen more effective features. At the same time, the distribution of the EEG covariance matrix is unified in Riemann space, and then a domain adaptive method is extended to realize end-to-end adaptation in capsule decision neural networks. CDNN can effectively fuse the EEG features extracted from primary capsules, effectively reduce the loss of feature information, and improve the robustness of small sample learning. Experiments on two representative motor imagery EEG datasets verify the effectiveness of CDNN.

At the same time, the proposed capsule neural decision network embodies a bioinspired paradigm, reflecting the hierarchical information processing mechanism observed in the biological neural system. Inspired by the columnar tissue of the cerebral cortex and the dynamic routing of sensory information, each capsule carrier in our architecture represents a special collection of neurons, encoding the spatiotemporal pattern of motor imagery. The proposed method simulates the predictive coding strategy of the brain through neural decision-making routing and realizes the decoding of the imaginary motion in the EEG signal, which has the adaptability of imitating human beings. This bionic method not only enhances the robustness of classification, but it also promotes the development of the brain–computer interface by establishing the symbiotic relationship between neurophysiological insights and artificial intelligence (the cornerstone of modern bionic engineering).

The innovation of the method proposed in this article can be summarized as follows:

1. This article innovatively constructs a deep capsule decision network by introducing multiple primary capsules to form a hidden layer and using neural decision routing algorithms to dynamically determine the connection between advanced capsules and primary capsules. This mechanism not only enhances the model’s ability to capture complex relationships between features, but it also improves the flexibility and accuracy of feature selection through probabilistic decision-making, effectively avoiding potential local optima problems in traditional routing algorithms.
2. In response to the high-dimensional and non-Euclidean spatial characteristics of EEG data, this paper integrates a method of aligning the distribution of the EEG covariance matrix in Riemannian space. Not only does it reduce the loss of feature information, but it also enables the model to more naturally process the intrinsic geometric structure of EEG data.
3. By using the extended domain adaptation method, end-to-end adaptation of the capsule decision neural network is achieved, improving the model’s generalization ability under different datasets and experimental conditions, as well as its robustness and accuracy in small-sample learning scenarios.

The rest of this paper is organized as follows: the second section introduces Riemann alignment, neural decision forest, capsule neural network, and other related works. The third section describes the details of the capsule decision neural network based on transfer learning (CDNN) proposed in this paper. The fourth section introduces the experiment of comparing the performance of CDNN with several representative data alignment and transfer learning methods. Finally, the fifth section summarizes this paper.

## 2. Related Work

### 2.1. Riemann Alignment (RA)

EEG is essentially a spatiotemporal sequence recorded by multi-channel electrodes, and its correlation covariance matrix between channels is naturally located in the Riemannian manifold. The traditional Euclidean space method loses the geometric structure information. Riemann alignment maps the EEG signals of different subjects into a unified geometric framework through geometric transformations on manifolds, such as tangent space projection and parallel transmission, to eliminate individual specificity and retain the intrinsic characteristics of neural activity. The key steps of Riemann alignment are as follows [22]:

First, the covariance matrix $P_{i=1}^{n}$ that captures the spatial correlation between channels is calculated, and then the Riemann mean$\;M_{R}$ of all subjects is calculated through iteration. The Riemann mean is the “geometric center” of all points on the manifold. Each covariance matrix $P_{i=1}^{n}$ is projected onto the tangent space of the Riemann mean$\;M_{R}$ (local linear approximation) to eliminate the influence of manifold curvature (such as individual differences between subjects), as shown in the following Formula (1):

(1) $$P_{i}^{\prime }=M_{R}^{-1/2}P_{i}M_{R}^{-1/2}$$

where $P_{i}$ is the covariance matrix of the $i^{\mathrm{th}}$ sample, and $P_{i}^{\prime }$ is the covariance matrix obtained after $P_{i}$ Riemann alignment [8].

### 2.2. Neural Decision Forest

In reference [29], a differentiable decision forest model, namely the neural decision forest model, was proposed by combining decision forest with an artificial neural network. The task of neural decision forest learning is to reduce the randomness of tree node routing decisions to transform the task of representing learning into a minimization loss function. The greatest contribution of neural decision forest is to build a bridge between traditional machine learning and deep learning that can fully combine their respective advantages. Neural decision forest models have obvious advantages in learning tasks with few samples.

### 2.3. Capsule Neural Network

A capsule network is a new neural network structure proposed by Hinton et al. [30]. It solves the limitation of traditional CNN in spatial hierarchical modeling. It uses primary and advanced capsules to capture the pose and deformation of objects. The primary capsule is located in the shallow layer of the capsule neural network, and local features are extracted from the input image by convolution operation. Each primary capsule is actually a group of neurons that represents the feature attributes in the form of vectors. The advanced capsule is located in the deep layer. It integrates the primary capsule information through the routing mechanism to capture complex objects. The vector length represents the probability of object existence. Through dynamic routing, the capsule learns the local global relationship and improves the robustness of identifying complex structures. A capsule network can handle tasks that require an understanding of the target direction and spatial context, and it provides a feasible method to solve the problem of computer vision.

## 3. Materials and Methods

### 3.1. Dataset

The datasets used in this experiment are two motor imagery EEG datasets, and their detailed statistical data are shown in Table 1.

The motor imagery EEG dataset MI1 recorded the motor imagery EEG experiments of 7 subjects [31], including 200 groups of experiments (100 groups of left-hand motor imagery and 100 groups of right-hand motor imagery). The EEG signals of 59 channels were recorded in the experiment, and the sampling frequency was 100 Hz.

**Figure 1:**
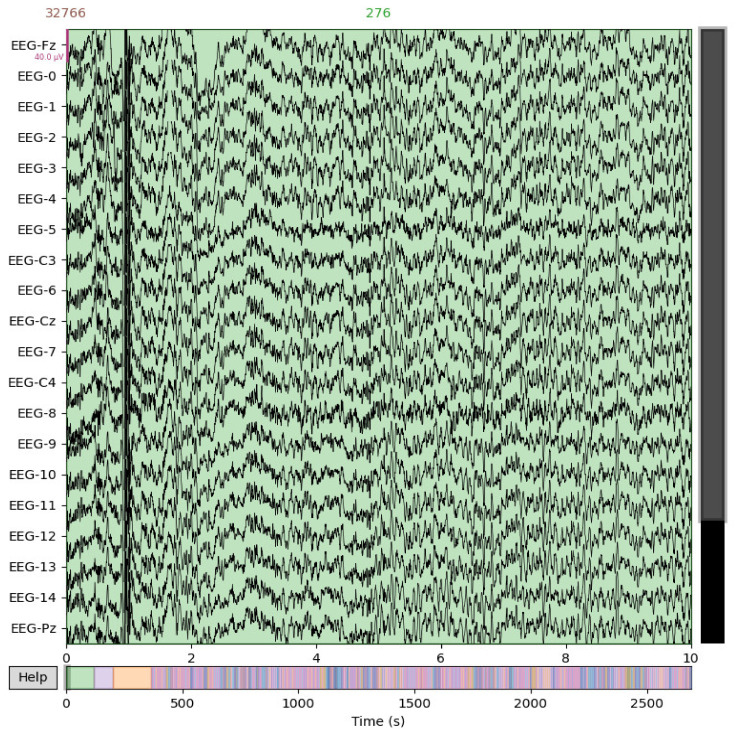
Schematic representation of EEG signals from dataset MI2.

**Figure 2:**
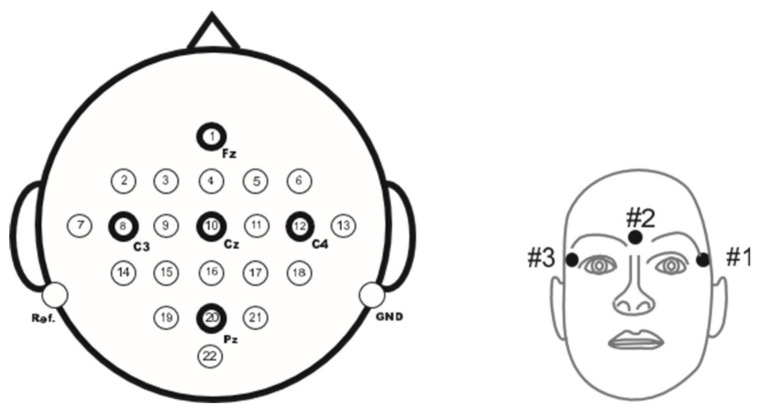
Left: Position distribution of 22 electrodes corresponding to the international 10–20 system. Right: Position distribution of 3 electrodes for recording electrooculography.

The motor imagery EEG dataset MI2 recorded the motor imagery EEG experiments of 9 subjects [32], including 144 groups of experiments (72 groups of left-hand motor imagery and 72 groups of right-hand motor imagery). The EEG signals of 22 channels were recorded in the experiment, and the sampling frequency was 250 Hz. Figure 1 shows the EEG signal of a sample in dataset MI2. The position distribution map of 22 electrodes is shown in the left subplot of Figure 2, while the right subplot of Figure 2 records the position distribution map of 3 electrodes in the electro analyzer (EOG).

This paper chose these two datasets to evaluate the proposed method because they are open datasets that are highly standardized, multi-subject, multi-sample, challenging, and suitable for evaluating classification models.

### 3.2. Transfer Learning and Capsule Decision Neural Network (CDNN)

**Figure 3:**
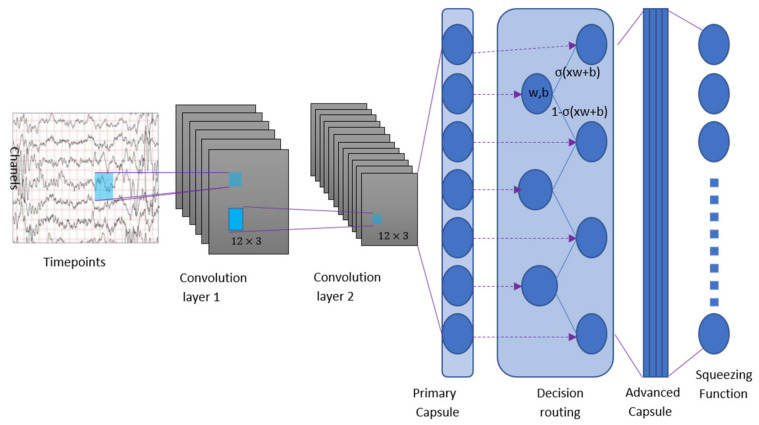
Capsule decision neural network based on transfer learning.

Figure 3 shows the architecture-proposed capsule decision neural network based on transfer learning (CDNN) in this paper. The architecture has only two convolution layers and a fully connected layer, which is a shallow neural network. Convolutional layer 1 has 64 12×3 convolution kernels with a stride of 1, and RELU is activated. This layer converts the EEG signal strength into the activity of the local feature detector, and then uses it as the input of the primary EEG capsule.

The primary EEG capsule has the lowest level of multidimensional features. From the perspective of reverse features, activating the primary EEG capsule corresponds to the reverse rendering process. The primary capsule layer is a capsule layer composed of a convolution neural network layer, with 150 convolution 8D capsule channels (that is, each primary capsule contains 8 convolution units, with 12 × 3 cores and 1 step). In general, primary capsules have a capsule output of 150 (each output is an 8D vector), and each 12 × 3 capsule in the mesh shares its weight with the others. The primary capsule is essentially a grouping convolution, and the “squeezing function” can be regarded as a nonlinear transformation unit.

The EEG category capsule in the last layer (class-caps) is a 16 dimensional capsule, and each EEG category capsule receives input from all primary capsules on the upper layer. The capsule decision-making neural network proposed in this paper uses probabilistic decision-making to realize the information exchange from primary EEG capsule to EEG category capsule.

The length of the capsule represents the probability that the relevant EEG features appear in the current input. The length of the capsule can be constrained by the extrusion function so that the short capsule tends to 0 and the long capsule tends to 1:

(2) $$v_{j}=\frac{\left||c_{j}|\right|}{1+||c_{j}|{|}^{2}}\frac{c_{j}}{\left||c_{j}|\right|}$$

where $v_{j}$ is the vector output of capsule j, and $c_{j}$ is the output from the primary EEG capsule.

In the CDNN algorithm, the characteristic capsule output from the capsule layer is classified by the decision forest. For each decision tree, the feature capsule follows the routing algorithm to reach the capsule on the leaf node through the decision tree to make a prediction. In this network, the mapping from the neuron of the feature capsule layer to the decision node is shown as follows:

(3) $$d_{n}(v_{j};\theta )=\sigma (f_{n}(v_{j};\theta ))$$

where $v_{j}$ represents input, θ is a parameter, σ is a sigmoid function, and f(⋅;θ) is a real valued function depending on input $V_{j}$ and parameter θ. Through the above formula, the mapping from feature capsule layer to decision node is realized. If the feature capsule wants to reach the leaf node through the tree, it needs to plan the route.

A problem of EEG signal classification is investigated, in which the feature capsule is the input space V and the EEG category capsule is the output space Y. Decision tree is a tree structure classifier composed of decision (or split) nodes and prediction (or leaf) nodes. The decision node of the N index is the internal node of the tree, and the prediction node of the L index is the terminal node of the tree.

Each prediction node l∈L maintains a probability distribution π on the category capsule output space Y, and each decision node n∈N is assigned a decision function $d_{n}(\;\cdot \;;\Omega ):V_{j}\to [0,1]$ by Ω parameterization, which is responsible for routing feature capsules along the tree.

When the feature capsule $v_{j}\in V$ reaches the decision node n, it will be sent to the left subtree or the right subtree according to the output of $d_{n}(v_{j};\Omega )$. In the standard decision forest, $d_{n}$ is binary, and the route is deterministic. This paper considers probabilistic routing; that is, the routing direction is the output of the Bernoulli random variable of average $d_{n}(V_{j};\Omega )$. When the sample ends at the leaf node, the relevant tree prediction is given by class label distribution π. In the case of random routing, the category capsule is obtained at the leaf node, and the category probability capsule is normalized by the squeezing function. Therefore, the final prediction of the characteristic capsule by the tree T whose point parameter is a decision node is given by the following formula:

(4) $$P_{T}[y|v_{j},\Omega ,\vec{\pi }]=\sum \pi_{ly}\mu_{l}(v_{j},\Omega )$$

In formula $\pi ={\left(\pi_{l}\right)}_{l\in L}$, $\pi_{ly}$ represents the probability that the feature capsule $v_{j}$ reaches the leaf node L and belongs to the category capsule y, and $\mu_{l}(v_{j}|\Omega )$ is the probability routing function of the feature capsule $v_{j}$ reaching the leaf. The decision routing function is $\mu (v_{j}|\Omega )$, and Boolean variables $b_{l}$ and $b_{r}$ are introduced. If the capsule is routed to the left subtree, then $b_{l}$ is true; otherwise, $b_{r}$ is true. $\mu (v_{j}|\Omega )$ can be expressed as follows:

(5) $$\mu (v_{j}|\Omega )={\prod_{n\in Ν}d_{n}(v_{j};\Omega )}^{b_{l}}\bar{d_{n}}{\left(v_{j};\Omega \right)}^{b_{r}}$$

where $\bar{d_{n}}(v_{j};\Omega )=1-d_{n}(v_{j};\Omega )$, and $b_{l}$ and $b_{r}$ are bool variables.

### 3.3. Decision Node

In this paper, we consider the random routing of the decision function transfer class capsule. The definition of the decision function is as follows:

(6) $$d_{n}(v_{j};\Omega )=\sigma (f_{n}(v_{j};\Omega ))$$

where $\sigma (x)=(1+e^{x})-1$ is a sigmoid function and $f_{n}(v_{j};\Omega )$ is a real valued function, which depends on the feature capsule $v_{j}$ and parameter Ω as input.

### 3.4. Back Propagation Training Network

We can train the capsule decision network through error back propagation. The main task of training is to estimate the decision node parameter Ω and leaf decision vector π under a given training set T⊆X×Y:

(7) $$R(\Omega ,\pi ;Τ)=\frac{1}{\left|Τ\right|}\sum_{(x,y)\in Τ}L(\Omega ,\pi ;x,y)$$

where L(Ω,π;x,y) is the logarithmic loss term of training sample (x,y)∈T:

(8) $$L(\Omega ,\pi ;x,y)=-\log(P_{Τ}[y|v_{j},\Omega ,\pi )$$

$P_{Τ}$ is defined by Formula (4). At the same time, in order to reduce the distribution difference between the target domain and the source domain, we introduce adaptive loss, which is defined as the distance between the covariance of the source feature and the target feature:

(9) $$l_{A}=\frac{1}{4d^{2}}||P_{S}^{\prime }-P_{T}^{\prime }|{|}_{F}^{2}$$

where ${\left|\left|\cdot \right|\right|}_{F}^{2}\;$represents the Frobenius norm of the square matrix. $P_{S}^{\prime }$ and $P_{T}^{\prime }$ are the covariance matrix of source data and target data after the Riemann alignment (see Formula (1)). The covariance matrix of the source data and target data is derived from the following formula:

(10) $$P_{S}^{\prime }=\frac{1}{n_{S}-1}\left(D_{S}^{T}D_{S}-\frac{1}{n_{S}}{\left(1^{T}D_{S}\right)}^{T}\left(1^{T}D_{S}\right)\right)$$

(11) $$P_{T}^{\prime }=\frac{1}{n_{T}-1}\left(D_{T}^{T}D_{T}-\frac{1}{n_{T}}{\left(1^{T}D_{T}\right)}^{T}\left(1^{T}D_{T}\right)\right)$$

where 1 is the column vector where all elements are equal to 1. The gradient of input features can be calculated using chain rules:

(12) $$\frac{\partial l_{A}}{\partial D_{S}^{ij}}=\frac{1}{d^{2}\left(n_{S}-1\right)}{\left({\left(D_{S}^{T}-\frac{1}{n_{S}}{\left(1^{T}D_{S}\right)}^{T}1^{T}\right)}^{T}\left(P_{S}^{\prime }-P_{T}^{\prime }\right)\right)}^{ij}$$

We describe our method with an example of the problem of two classifications of EEG signals. The transportability of ordinary neural networks is relatively limited, which can easily cause over fitting from the target domain, resulting in a significant decline in the classification performance of the target domain or even complete failure. Therefore, we can use the joint training of classification loss and domain adaptation alignment loss to realize the transfer from source domain to target domain.

(13) $$L_{TOTAL}=L\left(\Omega ,\pi ;x,y\right)+\sum_{i=1}^{c}\eta_{i}l_{A}$$

where c represents the number of domain adaptive alignment loss layers in the capsule decision neural network, and η is the weight of adaptive loss.

### 3.5. Summary of Learning Procedures

Algorithm 1 summarizes the learning process. Starting with the random initialization of the parameter Ω of the decision node, we iterate the learning process for a predetermined number of times given the training set T. In each round, we first generate feature capsules, which form a small batch sequence. By running an iteration scheme, we obtain an estimate of the predicted leaf node parameter π for a given actual value Ω starting with the category capsules initialized to be evenly distributed on each leaf node. We then perform the SGD update for each sample.
**Algorithm 1:** Capsule decision neural network based on Transfer LearningInput: sample $X_{s}$ of source domain; Sample $X_{t}$ of target domain Output: target domain label vector ${\hat{y}}_{T}\in R^{n_{T}\times l}$, the labels for ${{\{X}_{T,i}\}}_{i=1}^{n_{T}}$. 1. Align the source domain covariance matrix to obtain ${{\{P}_{S,i}^{\prime }\}}_{i=1}^{n_{S}}$; 2. flat_prob = build_tree_projection(Category capsule) #Obtain the activation probability information of capsule neurons; 3. routes = build_routes(flat_prob) #Using probabilistic routing to calculate decision probability matrix; 4. features = concatenate (routes) #The decision probability matrix of M trees is merged into a big matrix; 5. leafs = build_Category # Calculate category probability matrix; 6. for 1:M do #The category capsule is calculated by matrix multiplication between decision probability matrix and category probability matrix; 7.  matmul(features,leafs)  8.   Squash; #Use the extrusion function to ensure that the length of the category capsule is in the interval [0, 1]; 9: end for  10. return ${\hat{y}}_{T}$.

The time complexity of the capsule decision neural network proposed in this paper mainly needs to consider the time complexity of the two stages of feature selection and hierarchical decision-making. The time complexity of feature selection by neural decision network is O(L⋅N⋅(D+K⋅M)), where N is the number of samples, and D is the feature dimension. K is the number of selected features, M is the hidden layer dimension, and it is assumed that the number of decision steps is L. The time complexity of hierarchical decision is $O(N\cdot C_{\mathrm{in}}\cdot C_{out}\cdot D_{in}\cdot D_{out})$. The total complexity of the proposed algorithm is $O(L\cdot N\cdot (D+K\cdot M)+N\cdot C_{\mathrm{in}}\cdot C_{out}\cdot D_{in}\cdot D_{out})$, where $C_{\mathrm{in}}$ and $C_{out}$ are the number of input and output capsules, and $D_{in}$ and $D_{out}$ are the capsule dimensions.

The time complexity of the original capsule network mainly needs to consider the capsule layer time complexity and dynamic routing time complexity: the time complexity of each capsule layer is $O(N\cdot C_{\mathrm{in}}\cdot C_{out}\cdot D_{in}\cdot D_{out})$, where $C_{\mathrm{in}}$ and $C_{out}$ are the number of input and output capsules, and $D_{in}$ and $D_{out}$ are the capsule dimensions.

The time complexity of dynamic routing is explained as follows: the number of iterations is R, the complexity of each iteration is $O(N\cdot C_{\mathrm{in}}\cdot C_{out}\cdot D_{in}\cdot D_{out})$, and the total complexity is $O(R\cdot N\cdot C_{\mathrm{in}}\cdot C_{out}\cdot D_{in}\cdot D_{out})$. The total time complexity of the algorithm proposed in this paper is slightly higher than that of the original capsule network, but the improved routing mechanism realizes the soft decision boundary through the neural decision network. Combined with the dynamic routing mechanism of the capsule network, it improves the classification ability of the model for complex data.

## 4. Results and Discussion

### 4.1. Experimental Configuration

In the process of model performance evaluation, we divided single-source domain transfer and multi-source domain transfer. Single-source domain transfer refers to using the test samples of a single subject as the training set, while correspondingly, multi-source domain transfer refers to using the test samples of multiple subjects as the training set.

For example, dataset MI2 includes nine subjects, so we have 9 × 8 = 72 single-source domain transfer learning tasks, such as S1 → S2 (subject 1 is the source domain, subject 2 is the target domain), S3 → S2, S4 → S2, S5 → S2, S6 → S2, S7 → S2, S8 → S9, S7 → S2, …, S8 → S9, etc. In the multi-source domain transfer (MTS) experiment, there are only nine transfer learning tasks. For example, {S2, S3, S4, S5, S6, S7, S8, S9} → S1, …, {S1, S2, S3, S4, S5, S6, S7, S8} → S9, etc.

In this study, balanced classification accuracy (BCA) was used to evaluate the classification accuracy of the proposed model. The calculation method of BCA is explained as follows: for each category, its recall rate is calculated, and the average recall rate of all categories is found. The advantage of BCA is that it assigns the same weight to each category, allowing for a fair evaluation of the model’s performance across all categories. The definition of BCA is shown in the following Formula (14):

(14) $$BCA=\frac{1}{N}\sum_{i=1}^{N}\frac{tp_{i}}{n_{i}}$$

where $tp_{i}$ and $n_{i}$ are the true positive number and the number of samples in the class, respectively.

At the same time, this article uses *p*-values for statistically significant testing to determine whether the observed data results are significantly different from the results that random errors can produce. If the *p*-value of a classification result is small (usually less than a certain significance level, such as 0.05), it indicates that the proposed model has a significant impact on classification performance; that is, the improvement of classification performance by the proposed model is effective.

### 4.2. Classification Accuracy

In order to further illustrate the superior generalization ability of CDNN (CDNN-R: Riemannian average is used as the reference matrix; CDNN-E: Euclidean average is used as the reference matrix; CDNN-L: logarithmic Euclidean average is used as the reference matrix) in transfer learning, its recognition accuracy is compared with relevant classical models such as CSP-LDA, EA-CSP-LDA, RA-MDM, CA, CA-Coral, CA-GFK, CA-JDA, CA-JGSA, and other methods [22,28,33,34,35,36].

EEG signal preprocessing has a crucial impact on all of the experimental results. In order to accurately evaluate the method proposed in this paper, we use the same EEG signal preprocessing method as the literature [28].

**Figure 4:**
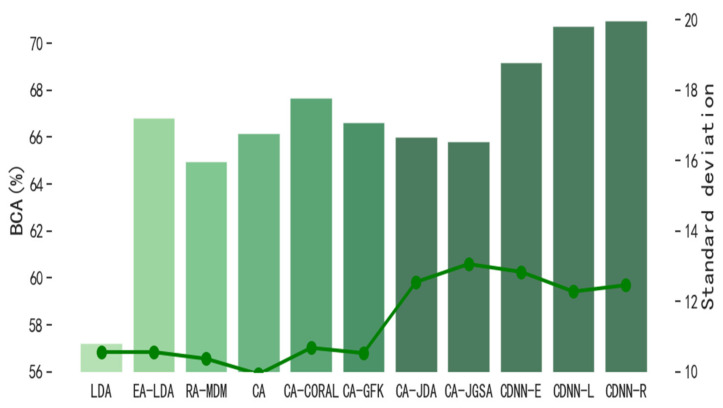
Average value of BCA in single-source domain transfer on dataset MI1 (%).

**Figure 5:**
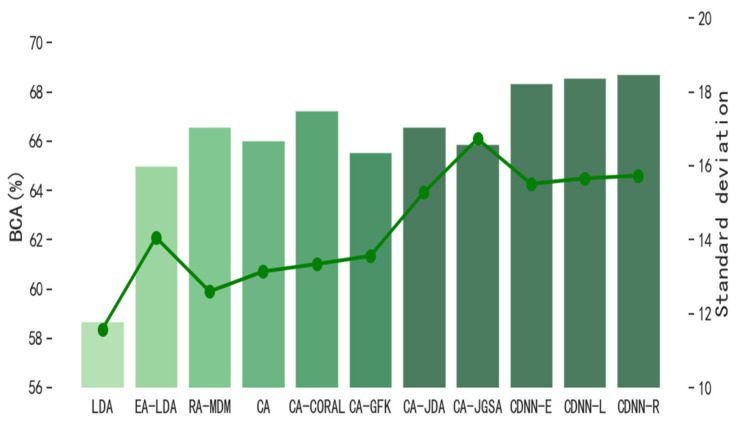
Average value of BCA in single-source domain transfer on dataset MI2 (%).

**Figure 6:**
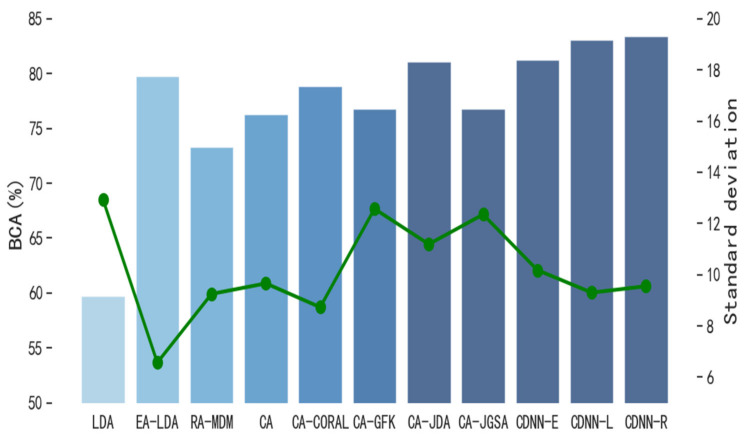
Average value of BCA in multi-source domain transfer on dataset MI1 (%).

**Figure 7:**
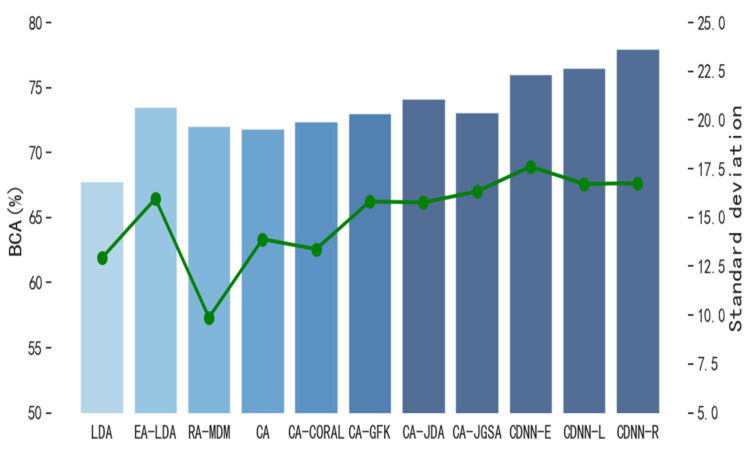
Average value of BCA in multi-source domain transfer on dataset MI2 (%).

In order to evaluate the performance of CDNN in single-source domain transfer learning, we compare CDNN with other classical transfer learning methods. The number M of hyper-parameter decision trees in the model is 3, and the number n of leaf nodes in each tree is 16. Figure 4 and Figure 5 show the performance of CDNN on the motion imagination dataset MI1 and MI2, respectively. At the same time, in order to evaluate the performance of CDNN in multi-source domain transfer learning, we also compare CDNN with other classical transfer learning methods. Figure 6 and Figure 7 show the performance of CDNN in multi-source domain transfer learning on motion imagination datasets MI1 and MI2, respectively. As shown in the figure, our method shows good generalization ability and satisfactory accuracy in both single-source domain transfer and multi-source domain transfer learning.

**Figure 8:**
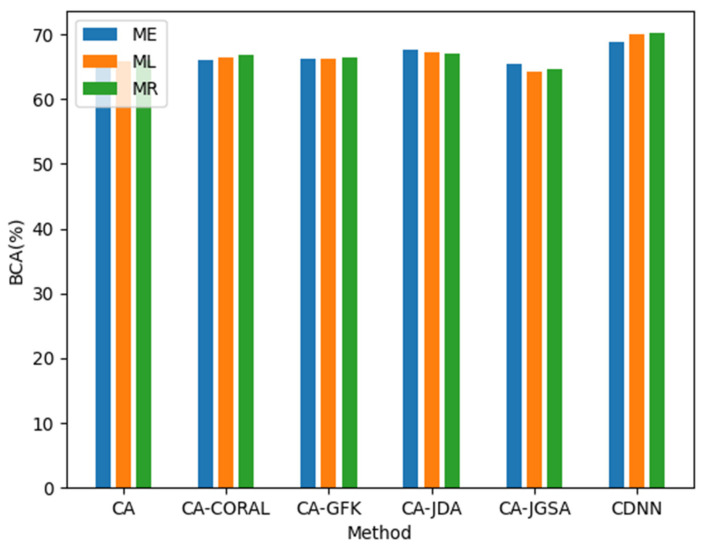
Average accuracy (%) of two motion imagery datasets using different reference matrices when aligning the source domain and the target domain.

In order to evaluate the influence of different reference matrices on the transfer from source domain to target domain, we compare the Riemann mean with the Euclidean mean (EM) and logarithmic mean (LM). Among the six transfer learning methods, including the CDNN proposed in this article, the Riemann mean has significant advantages, but its computational complexity is comparable to LM but greater than EM, requiring a balance between accuracy and speed, as shown in Figure 8.

These experiments show that the classification performance of the proposed model has certain advantages, which may be due to the capsule network through the following mechanisms to enhance the noise robustness and improve the performance of the model.

In hierarchical feature coding, capsule vectors (rather than scalar neurons) explicitly encode spatial hierarchical relationships (such as phase synchronization in brain regions) and have the ability to suppress local noise interference.

In a neural decision-making routing mechanism, the coupling coefficient is adjusted adaptively through neural decision-making to reduce the contribution weight of the noise capsule for the classification of decision-making.

Advantages of the representation of the module length of the capsule vector are that the module length of the capsule vector represents the signal strength and naturally suppresses the interference of small-amplitude noise.

In order to verify whether the performance improvement of CDNN-R is statistically significant, we conducted a *t*-test on the accuracy of the model, and the proposed model showed significant performance improvement (*p* < 0.05), as shown in Table 2. The selection of the parameter test method is based on the fact that the data obey normal distribution, and the sample size is sufficient. The results show that CDNN-R has obvious advantages in single-source domain transfer learning. In multi-source domain transfer learning, the performance improvement of the model is not obvious, which is understandable to some extent, because the performance difference of machine learning algorithms tends to decrease with the increase of training data.

Meanwhile, we further compared the performance of the proposed method with existing methods based on Riemannian manifolds in EEG classification tasks. In order to facilitate a comparison with existing methods based on Riemannian manifolds, we only selected five subjects (1, 2, 7, 8, and 9) from the BCI iv 2a dataset, and we excluded the other four subjects. In each experiment, one subject was selected as the target domain and the remaining subjects were used as the source domain. The baseline methods used for comparison include a parallel transport (PT) algorithm [23], transmissive parameter transfer (TPT) algorithm [28], coordination alignment algorithm based on Riemann space and a novel parameter transfer method (SPT), and PT-SPT [37]. The experimental results are shown in Table 3. From the table, we can see that, due to the alignment of EEG signals and the use of capsule decision neural network for further feature extraction and classification, it can effectively alleviate specific types of feature distortions, such as spatial information confusion, and it achieve better classification accuracy compared to baseline methods.

**Figure 9:**
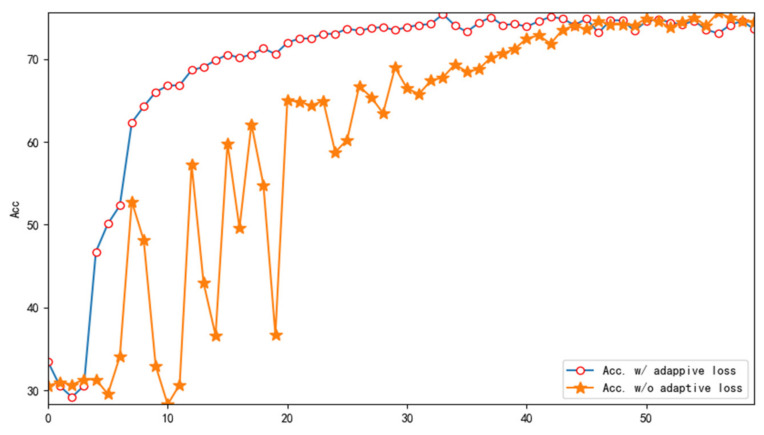
Accuracy with/without adaptive loss.

In order to verify the effect of introducing adaptive loss La on the performance of CDNN, we conducted ablation experiments. Figure 9 shows a comparison of the accuracy of the test (target domain) with and without adaptive loss $l_{A}$. From the figure, it can be seen that the introduction of adaptive loss improves the accuracy of the target domain.

In addition, we further conducted ablation experiments to test the effectiveness of the neural decision-making (ND) module in our proposed model. Table 4 shows the impact of removing neural decision blocks from the proposed model on the accuracy of multi-source domain transfer classification tasks for motor imagery. The dataset we used was MI2. The experimental results show that, benefitting from the implementation of decision trees using neural networks to avoid hard boundaries, the neural decision module improved the accuracy of the model by 1.3%.

## 5. Conclusions

This article proposes an innovative deep capsule decision network model aimed to solve the problem of feature distortion that is prone to occur during EEG feature extraction and improve the performance of the model in complex EEG data analysis. This model achieved this goal through the following key innovative points:

Deep capsule architecture and neural decision routing: We constructed a deep hidden layer consisting of multiple primary capsules, which utilizes neural decision routing algorithms to dynamically determine the connections between advanced capsules and primary capsules. This mechanism calculates the relationship between each capsule in the deep and shallow hidden layers in a probabilistic decision-making manner, effectively avoiding the limitations of traditional routing algorithms and improving the model’s ability to capture complex relationships between features and the flexibility of feature selection.

Data processing and domain adaptation in Riemannian space: In response to the high-dimensional and non-Euclidean characteristics of EEG data, we unified the distribution of the EEG covariance matrix in Riemannian space, enabling the model to more naturally handle the intrinsic geometric structure of EEG data. At the same time, by extending the domain adaptation method, end-to-end adaptation of the capsule decision neural network was achieved, further enhancing the model’s capture of EEG signal temporal dependence and spatial distribution characteristics, effectively reducing the loss of feature information and improving the model’s robustness and accuracy in small sample learning scenarios.

The experimental results show that the model has achieved significant performance improvement in both single-source domain transmission and multi-source domain transmission tasks on two representative motion image EEG datasets, verifying its effectiveness and practicality. This method provides not only new ideas and methods for EEG feature extraction and classification, but it also provides important references and inspirations for research in related fields. However, the proposed model may be limited by the depth of the decision tree, resulting in insufficient expression ability. The flexibility of static routing may also be weaker than that of dynamic iteration. The adaptability in more application scenarios needs to be further studied in future work.

## Figures and Tables

**Table 1:** Statistical data of motion imagination dataset MI1 and MI2.

Dataset	Number of Subjects	Number of Channels	Number of Time Samples	Trail per Subject
MI1	7	59	300	200
MI2	9	22	750	144

**Table 2:** *p* value in T-test.

The Ways of Transferring	CDNN-R vs	MI1	MI2
	CSP-LDA	0.0000	0.0000
	EA-CSP-LDA	0.0030	0.0002
	CSP-LDA	0.0000	0.0000
STS	CA-CORAL	0.0004	0.0250
	CA-GFK	0.0000	0.0001
	CA-JDA	0.0003	0.0124
	CA-JGSA	0.0018	0.0004
	CSP-LDA	0.0015	0.0043
	EA-CSP-LDA	0.0274	0.0152
	CA	0.0345	0.0136
MTS	CA-CORAL	0.0069	0.0173
	CA-GFK	0.0079	0.0135
	CA-JDA	0.0267	0.0125
	CA-JGSA	0.0145	0.0137

**Table 3:** Comparison of accuracy between the proposed model and the baseline Riemann classification method.

Methods	S1	S2	S7	S8	S9	Mean
TPT	0.4676	0.6389	0.3750	0.6343	0.4907	0.5290
PT	0.6296	0.6759	0.6528	0.7454	0.7917	0.6991
SPT	0.6574	0.7407	0.6806	0.7361	0.8102	0.7250
PT-SPT	0.6759	0.7129	0.6898	0.7500	0.7917	0.7241
Proposed	0.7176	0.7688	0.7591	0.7776	0.8246	0.7695

**Table 4:** The contribution of ND block in the proposed model to improve MI classification accuracy.

Removed Block	Accuracy	K-Score
None	78.79	0.714
ND	77.49	0.721

## Data Availability

The raw data supporting the conclusions of this article will be made available by the authors upon request.

## References

[1] Premchand B., Zhang Z., Ang K.K., Yu J., Tan I.O., Lam J.P.W., Choo A.X.Y., Sidarta A., Kwong P.W.H., Chung L.H.C. (2025). A Personalized Multimodal BCI–Soft Robotics System for Rehabilitating Upper Limb Function in Chronic Stroke Patients. Biomimetics.

[2] Ha J., Park S., Han Y., Kim L. (2025). Hybrid BCI for Meal-Assist Robot Using Dry-Type EEG and Pupillary Light Reflex. Biomimetics.

[3] He B., Baxter B., Edelman B.J., Cline C.C., Ye W.W. (2015). Noninvasive brain-computer interfaces based on sensorimotor rhythms. Proc. IEEE.

[4] Wei W., Qiu S., Zhang Y., Mao J., He H. (2022). ERP prototypical matching net: A meta-learning method for zero-calibration RSVP-based image retrieval. J. Neural Eng..

[5] Sun J., Wei M., Luo N., Li Z., Wang H. (2022). Euler common spatial patterns for EEG classification. Med. Biol. Eng. Comput..

[6] Blanco-Diaz C.F., Antelis J.M., Ruiz-Olaya A.F. (2022). Comparative analysis of spectral and temporal combinations in CSP-based methods for decoding hand motor imagery tasks. J. Neurosci. Methods.

[7] Lei C., Zheng S., Zhang X., Wang D., Wu H., Peng H., Hu B. (2022). Epileptic Seizure Detection in EEG Signals Using Discriminative Stein Kernel-Based Sparse Representation. IEEE Trans. Instrum. Meas..

[8] Barachant A., Bonnet S., Congedo M., Jutten C. (2012). Multiclass braincomputer interface classification by Riemannian geometry. IEEE Trans. Biomed. Eng..

[9] Yger F., Berar M., Lotte F. (2017). Riemannian approaches in braincomputer interfaces: A review. IEEE Trans. Neural Syst. Rehabil. Eng..

[10] Tang S., Liu C., Zhang Q., Gu H., Li X., Li Z. (2021). Mental workload classification based on ignored auditory probes and spatial covariance. J. Neural Eng..

[11] Cai Z., Wang L., Guo M., Xu G., Guo L., Li Y. (2022). From Intricacy to Conciseness: A Progressive Transfer Strategy for EEG-Based Cross-Subject Emotion Recognition. Int. J. Neural Syst..

[12] Liu B., Chen X., Li X., Wang Y., Gao X., Gao S. (2021). Align and pool for EEG headset domain adaptation (ALPHA) to facilitate dry electrode based SSVEP-BCI. IEEE Trans. Biomed. Eng..

[13] Roy A.M. (2022). An efficient multi-scale CNN model with intrinsic feature integration for motor imagery EEG subject classification in brain-machine interfaces. Biomed. Signal Process. Control.

[14] Roy A.M. (2022). Adaptive transfer learning-based multiscale feature fused deep convolutional neural network for EEG MI multiclassification in brain-computer interface. Eng. Appl. Artif. Intell..

[15] Sharma R., Kim M., Gupta A. (2022). Motor imagery classification in brain-machine interface with machine learning algorithms: Classical approach to multi-layer perceptron model. Biomed. Signal Process. Control.

[16] Wu D., Lance B.J., Parsons T.D. (2013). Collaborative Filtering for Brain-Computer Interaction Using Transfer Learning and Active Class Selection. PLoS ONE.

[17] Kalashami M.P., Pedram M.M., Sadr H. (2022). EEG Feature Extraction and Data Augmentation in Emotion Recognition. Comput. Intell. Neurosci..

[18] Saberi Z.A., Sadr H., Yamaghani M.R. An Intelligent Diagnosis System for Predicting Coronary Heart Disease. Proceedings of the 2024 10th International Conference on Artificial Intelligence and Robotics.

[19] Moraes C.P.A., Fantinato D.G., Neves A. A Novel Approach for Transfer Learning Motor Imagery Classification Based on IVA. Proceedings of the 2023 31st European Signal Processing Conference (EUSIPCO).

[20] Li D., Shin H.-B., Yin K., Lee S.-W. Domain-Incremental Learning Framework for Continual Motor Imagery EEG Classification Task. Proceedings of the 2024 46th Annual International Conference of the IEEE Engineering in Medicine and Biology Society (EMBC).

[21] Jin Y., Mousavi M., de Sa V.R. Adaptive CSP with subspace alignment for subject-to-subject transfer in motor imagery brain-computer interfaces. Proceedings of the 6th International Conference on Brain-Computer Interface (BCI).

[22] Zanini P., Congedo M., Jutten C., Said S., Berthoumieu Y. (2018). Transfer learning: A Riemannian geometry framework with applications to braincomputer interfaces. IEEE Trans. Biomed. Eng..

[23] Yair O., Ben-Chen M., Talmon R. (2019). Parallel transport on the cone manifold of SPD matrices for domain adaptation. IEEE Trans. Signal Process..

[24] Sangineto E., Zen G., Ricci E., Sebe N. (2014). We are not all equal: Personalizing models for facial expression analysis with transductive parameter transfer. Proceedings of the 22nd ACM International Conference on Multimedia.

[25] Islam M., Lee T. Wavelet based Emotion Detection from Multi-channel EEG using a Hybrid CNN-LSTM Model. Proceedings of the TENCON 2022—2022 IEEE Region 10 Conference (TENCON).

[26] Veeranki Y.R., Mercado-Diaz L.R., Posada-Quintero H.F. Autoencoder Based Nonlinear Feature Extraction from EDA Signals for Emotion Recognition. Proceedings of the 2024 IEEE International Symposium on Medical Measurements and Applications (MeMeA).

[27] Veeranki Y.R., Posada-Quintero H.F. High-Resolution Time-Frequency Analysis of EEG Signals for Affective Computing. Proceedings of the 2024 46th Annual International Conference of the IEEE Engineering in Medicine and Biology Society (EMBC).

[28] He H., Wu D. (2020). Transfer learning for brain-computer interfaces: A Euclidean space data alignment approach. IEEE Trans. Biomed. Eng..

[29] Kontschieder P., Fiterau M., Criminisi A., Bulo S.R. Deep Neural Decision Forests. Proceedings of the IEEE International Conference on Computer Vision.

[30] Sara S., Frosst N., Hinton G.E. Dynamic Routing Between Capsules. Proceedings of the Neural Information Processing Systems.

[31] Data sets 1 ‹Motor Imagery, Uncued Classifier Application›. http://www.bbci.de/competition/iv/desc_1.html.

[32] Brunner C., Leeb R., Müller-Putz G., Schlögl A., Pfurtscheller G. BCI Competition 2008—Graz Data Set A. http://www.bbci.de/competition/iv/desc_2a.pdf.

[33] Sun B., Feng J., Saenko K. Return of frustratingly easy domain adaptation. Proceedings of the 30th AAAI Conference on Artificial Intelligence.

[34] Gong B., Shi Y., Sha F., Grauman K. Geodesic flow kernel for unsupervised domain adaptation. Proceedings of the IEEE Conference on Computer Vision and Pattern Recognition.

[35] Long M., Wang J., Ding G., Sun J., Yu P.S. Transfer feature learning with joint distribution adaptation. Proceedings of the IEEE International Conference on Computer Vision.

[36] Zhang J., Li W., Ogunbona P. Joint geometrical and statistical alignment for visual domain adaptation. Proceedings of the IEEE Conference on Computer Vision and Pattern Recognition.

[37] Tang X., Li X., Li W., Hao B., Xie Y., Dang X. (2021). Transfer Learning: Rotation Alignment with Riemannian Mean for Brain-Computer Interfaces and Wheelchair Control. IEEE Trans. Cogn. Dev. Syst..

